# Assessing the Efficacy of Broad-Spectrum Antibiotics in Controlling Bacterial Contamination in the *In Vitro* Micropropagation of Ginger (*Zingiber officinale* Rosc)

**DOI:** 10.1155/2020/6431301

**Published:** 2020-06-13

**Authors:** Selam Tewelde, Subban Patharajan, Zenebe Teka, Desta Berhe Sbhatu

**Affiliations:** ^1^Tigrai Biotechnology Center Pvt. Ltd. Co., Mekelle, Ethiopia; ^2^Aksum University, P.O. Box 1010, Aksum, Ethiopia; ^3^Mekelle University, P.O. Box 1632/231, Mekelle, Ethiopia

## Abstract

Ginger (*Zingiber officinale* Rosc) (Zingiberaceae) is a livelihood and commercial crop in Ethiopia. But, the availability of clean and healthy planting materials has become a problem due to wilt disease, caused by *Ralstonia solanacearum* Biovar 3 Race 4. This problem obliged growers to seek for tens of millions of vigorous and disease-free planting materials very quickly via *in vitro* micropropagation of shoot tip explants. For this purpose, protocols of sterilizing shoot tip explants and controlling bacterial contamination of one Ethiopian ginger cultivar called Deribo were tested. Hence, this article reports the finding of a study that aimed at testing the (a) effectiveness of three sterilization agents, namely, 0.25% w/v RBK (composed of ridomile, bayleton, and kocide at 1 : 1 : 1 ratio), 0.50% v/v NaOCl, and 70% v/v ethanol at three different treatment times in combination with 0.25% HgCl_2_; (b) efficacy of four broad-spectrum antibiotics and their combinations in controlling bacterial contaminants of ginger shoot tip explants and *in vitro* micropropagation media; and (c) effects of the antibiotics on the shooting performances of the explants of the cultivar. A 0.50% v/v NaOCl at exposure time of 20 min followed by 0.25% HgCl_2_ has resulted in 80% contamination-free and 70% live explants after three weeks of incubation. Likewise, cefotaxime at 50, 100, and 200 mg/L and cefotaxime plus streptomycin at 25, 50, and 100 mg/L yielded 87 to 93% contamination-free microshoots after three weeks of culturing. The number of explants killed by the antibiotics increased with increasing the concentration of the antibiotics. Cefotaxime at 50 mg/L and cefotaxime plus streptomycin at 25 mg/L yielded significantly highest mean microshoots per explant (7.10 ± 0.36 and 7.51 ± 0.27, respectively) and mean shoot length (4.2 ± 0.26 and 3.56 ± 0.17 cm, respectively). Some of the microshoots showed some yellowing. But, they turned green and grew normal after subcultured into fresh, antibiotics-free culture media. These findings are important foundations towards developing more optimized protocols of sterilizing explants and controlling bacterial contaminants for large-scale *in vitro* micropropagation of the Deribo ginger cultivar.

## 1. Introduction

Ginger (*Zingiber officinale* Rosc) (Zingiberaceae) is one of the world's most important medicinal and culinary spice plant [1]. It is a perennial herbaceous plant but cultivated as an annual crop. The plant has an erect stem covered with leaf sheaths, 15–20 cm long and 2-3 cm wide alternate leaves, a laterally compressed and palmately branched underground stem called rhizome, and fine fibrous roots [2]. Both fresh and dried rhizomes of ginger are valuable all over the world as spice and herbal medicine [3–5]. The plant is, thus, commercially cultivated in several countries including China, Fiji, India, Indonesia, Jamaica, Japan, Mexico, Philippines, and Taiwan, as well as in many African countries [6]. The crop thrives best in warm and humid climates from the sea level to 1,600 meters, with a mean temperature between 20 and 32°C and total annual rainfall of 1500 to 3000 mm [7]. It performs well in well-drained, fertile, and friable soil with a neutral pH [8].

Multiple varieties of ginger are extensively cultivated in southern and western Ethiopia. The plant is the principal livelihood crop in southern Ethiopia [9] covering about 85% of the arable land of the Southern Nations, Nationalities, and Peoples' State of Ethiopia [10]. It is a principal cash crop for small-scale farmers and one of the important commodities for local small-scale traders and wholesellers. Ginger is the most common spice in almost all Ethiopian traditional dishes. It is also used in the preparation of hot drinks and remedies. Currently, it is one of the most valued export commodities for Ethiopia [9, 11].

Being one of the 45 Ethiopian ginger cultivars, Deribo is fibrous and more pungent with higher content of volatile oil compared to the Asian ginger variety. Its taste is similar to a Nigerian ginger variety and is well-accepted in western cuisine. Ginger has been Ethiopia's number one export spice till 2013 [12]. It is given special focus in the Ethiopian agricultural transformation and export trade plan. Unfortunately, bacterial wilt is becoming a serious threat to ginger production and productivity in the country. The disease started spreading throughout Ethiopia since 2014, and producers have reported up to 98% yield loss in 2014 [11, 12]. The causative agent of the wilt is *Ralstonia solanacearum* Biovar 3 Race 4. It is already distributed throughout all ginger growing areas by recycling of seed rhizomes as the growers were neither aware about it nor have the knowledge to control its spread [11].

This national crisis requires the development of some strategy. The pathogen is soil-borne bacteria. Thus, not only the rhizomes are infected but also the ginger-growing soils are contaminated with the pathogen. The rhizomes often accumulate high dose of the pathogen and become unsuitable as planting materials. This calls for some dependable strategy aiming at producing vigorous, disease-free planting materials. In fact, there has been a growing demand for clean and healthy planting materials of improved and adapted varieties [13]. Large-scale *in vitro* micropropagation of the crop becomes the only viable strategy for mitigating the problem quickly and extensively. Tigrai Biotechnology Center Pvt. Ltd. Co., located in Mekelle (Ethiopia), is the only facility capable of producing millions of disease-free plantlets in few years. It can produce up to 40 million plantlets per annum. Currently, the facility is carrying out large-scale *in vitro* micropropagation of three cultivars of ginger, namely, Boziab, Deribo, and Yali.

However, the success of large-scale *in vitro* micropropagation principally depends on developing a good protocol, including the development of effective protocol of explant and media sterilization. Growth media and plantlets contamination is the main cause of loss in large-scale micropropagation facilities. While bacterial contamination of media can be controlled by autoclaving, the contamination of plantlets is very difficult to control, especially if the contaminants are endophytic such as the causative agent of ginger wilt disease [14]. Explants derived from Ethiopian ginger cultivars are highly unlikely to be free of the *R. solanacearum* Biovar 3 Race 4 bacteria. Moreover, surface sterilization can only clean the bacteria off the explants. Thus, the formulation of the growth media has to be enriched with chemical agents or antibiotics that kill the pathogenic bacteria. The use of broad-spectrum antibiotics such as streptomycin, kanamycin, cefotaxime, and gentamicin to enrich *in vitro* micropropagation media is a preferred procedure [15–17].

Clonal propagation of ginger is a well-established technology throughout the world. Shoot tips, axillary buds, root tips, rhizome axillary buds, vegetative buds, sprouting buds, young buds, and rhizome buds have been used as explants for *in vitro* regeneration of many ginger varieties successfully [3, 5, 6, 13, 18–21]. Likewise, clonal propagation of many Ethiopian ginger cultivars was attempted successfully using rhizome buds, shoot tips, and others sources of explants [10, 13]. However, explants derived from rhizome buds and similar structures produce limited number of offshoots per year. Clonal propagation using rhizome buds and other vegetative structures is not helpful in producing millions of planting materials quickly [19]. Shoot tips and shootlets or microshoots derived from rhizome buds are good choices as explants for rapid, large-scale *in vitro* micropropagation of the plant to alleviate this problem [6, 13, 19]. However, ginger shoot tips are not free of the pathogenic bacteria discussed above and many other contaminants. In fact, the technicians at the Tigrai Biotechnology Center have observed that *in vitro* cultures of Deribo cultivar are especially vulnerable to bacterial contamination. Therefore, this article reports the results of a study that attempted to develop a protocol of controlling bacterial contamination using three disinfectants and four broad-spectrum antibiotics for the *in vitro* micropropagation of the Ethiopian ginger cultivar of Deribo through shoot tip explants.

## 2. Materials and Methods

The experiment was conducted at the Tigrai Biotechnology Center Pvt. Ltd. Co. Lab, formerly known as Mekelle Plant Tissue Culture Laboratory. The center is located in Mekelle, Tigrai, Ethiopia (alt.: 1979 masl; lat.: 13°30″0′N; long.: 39°28″11′E) about 200 km southeast of the historic city of Aksum. It has the capacity of producing more than 40 million plantlets per annum.

### 2.1. Collection of Mother Plants and Explants

Mother plants (i.e., rhizomes) of the Deribo ginger cultivar (*Z. officinale* Rosc) used in this study were collected from the Hadiya and Kembata areas of the Southern Nations, Nationalities, and Peoples' State of Ethiopia. Vigorous rhizomes were selected and were sprayed with a mixture of ridomile, bayleton, and kocide. Then, they were put in moist coco peat to sprout and maintained in greenhouse for several weeks. Then, 90 shoot tips (2-3 cm long) were collected by excising from sprouted rhizomes as explants for *in vitro* micropropagation.

### 2.2. Preparation and Sterilization of Explants

Shoot tip explants were washed thoroughly with tap water and soap solution to remove traces of any dirt particles. Then, they were washed with sterile distilled water until all traces of the soap solution were removed and were readied for treatment. The study was designed to test the effectiveness of three surface sterilization agents, namely, 0.25% v/w RBK (lab-made antimicrobial agent composed of ridomile, bayleton, and kocide at a ratio of 1 : 1 : 1) (3 treatments), 70% v/v ethanol (3 treatments), and 0.50% v/v NaOCl (sodium hypochlorite) (3 treatment) in combination with of 0.25% HgCl_2_ (mercuric chloride). Surface sterilization using 0.25% RBK involved the treatment of the explants with the agent for 30, 45, and 60 minutes. Sterilization procedure using 0.50% NaOCl involved the treatment of explants for 10, 20, and 30 min. Sterilization using 70% ethanol involved treatment of the explants for 5, 10, and 15 min. In all cases, the explants were surface sterilized with 0.25% HgCl_2_ for 10 more min under the laminar airflow cabinet. Finally, the explants were washed with double distilled water to remove any traces of the HgCl_2_ and were readied for inoculation.

### 2.3. Preparation of *In Vitro* Culture Media

The study used Murashige and Skoog [22] (MS) media for *in vitro* culturing of the explants. Full-strength MS media were prepared by adding required stock solutions of micronutrients, macronutrients, and additives and by enriching with 30 g of sucrose (C-source) and 5 g of agar as solidifying agent. The pH of the solutions was adjusted to about 5.8 by adding drops of 0.1 N HCl and 0.1 N NaOH as appropriate. The content was heated at 90°C. Required number of initiation and shooting media were prepared by readying 40 mL culture media in 300 mL magenta culture bottles. Whilethe MS media assigned for initiation were enriched with 2.0 mg/L (BAP), those assigned for shooting were enriched with 4.0 mg/L BAP. All treatments were prepared in three replications. Finally, the media were autoclaved at 121°C and 15 psi for 20 min. While the initiation media were allowed to cool at room temperature to 60°C, the shooting media were enriched with antibiotics while cooling. The required concentrations and combinations of antibiotics were added to shooting media while they were cooling but not solidified. Finally, they were kept for a week to inspect their sterility and fitness for use. Some media were left without antibiotics to serve as control. Four broad-spectrum antibiotics, namely streptomycin, kanamycin, gentamicin, and cefotaxime were used alone at the rate of 50, 100, and 200 mg/L, as well as in possible combinations of two antibiotics at 25, 50, and 100 mg/L [15].

### 2.4. Inoculation and Incubation of Explants

The initiation and shooting media were inoculated with tip shoot explants at the rate of one shoot per bottle under a laminar airflow cabinet. Culture bottles of the treatments were placed on growth racks in a completely randomized design. The growth room was adjusted to 25 ± 2°C under a fluorescent tube light with 16/8 hours light/dark photoperiod and a light intensity of 1,200 lux. The shoot tip explants were incubated for three weeks. The explants were subcultured into fresh media twice in the interval of three weeks to produce enough microshoots for the shooting experiment. The inoculation and incubation of the shooting media were carried out with the same procedure except five microshoots which were put into each culture bottle holding shooting media.

### 2.5. Data Sources and Analyses

Quantitative data were the explant survival rate and rate of contamination of explants in initiation and shooting media, number of shoots per explant, and shoot length. Qualitative data included color and gross morphology of shoots. The data were collected after three weeks of incubation for initiation and shooting. Treatment effects in all tests were determined by using the analysis of variance (ANOVA) using the statistical package for social science (SPSS version 20) and means were compared using the Least Significant Difference (LSD). All comparisons were made at *a priori* significance level of *p* ≤ 0.05.

## 3. Results and Discussion

### 3.1. The Effects of Surface Sterilizers for Ginger Shoot Explants

The purpose of this study was to develop a standard procedure of surface sterilization of explants of the Deribo Ethiopian ginger cultivar and its *in vitro* micropropagation media. For this purpose, the effects of three sterilization agents with three exposure (treatment) times were tested. Sodium hypochlorite (0.50% v/v) at 20 min treatment time yielded a significantly highest mean number (8.00 ± 1.73) of clean explants after three weeks of incubation (Table 1; *p* ≤ 0.05). The treatment yielded 80% clean explants. NaOCl at 20 and 30 min treatment and 70% ethanol at 5 and 10 min treatment produced the highest mean numbers of live explants (7.33 ± 0.58 to 7.00 ± 1.00). But, the 70% ethanol (at all treatment times) was less effective in its disinfecting capacity.

Water (tap, distilled, and sterile), detergent solutions, antibacterial and antifungal agents, NaOCl, CaOCl_2_, ethanol, HgCl_2_, and antibiotics are the usual agents of sterilization of explants and controlling *in vitro* growth media contamination. The RBK detergent, NaOCl, ethanol, and HgCl_2_ are the most commonly used surface sterilization agents in preparing sterile explants for *in vitro* multiplication of many species and plant materials [23–28]. While the RBK, NaOCl, and ethanol are used as primary sterilization agents, the HgCl_2_ is often used as the secondary sterilization agent following the primary agents. In fact, some studies also showed that the HgCl_2_ can be effective when used alone [6, 29, 30]. But, their effectiveness might not show clear patterns. While Khatun et al. [29] observed 0.10% HgCl_2_ at 10 min exposure time to be the most effective, Suma et al. [30] observed the best sterilization result with the same concentration but shorter time in treating rhizome buds of ginger.

We looked into the effectiveness of the three primary sterilization agents in combination with HgCl_2_. The use of all these agents through some suitable procedures is more effective for sterilization than used singly [23–25]. But, simple disinfection or sterilization procedures save time and resources and cause less damage to the explants and, thus, are most preferred. This study showed that NaOCl (0.50% v/v) at 20 min treatment time in combination with HgCl_2_ at 10 min of treatment time yielded the best sterilization result. Similar results were reported with 20 min treatment time elsewhere [31]. Other researchers reported good sterilization results with lower concentration and exposure time of NaOCl [5, 29]. However, other workers showed that NaOCl at 10 min treatment time is less effective [6]. Such differences may be attributable to the genotypes of the plants or the types of explants. Anyway, care has to be taken that higher concentration of NaOCl (0.50% v/v), and other agents might cause some damage to the tissues of the explants to cause more contamination and death of explants [29, 32, 33].

The effectiveness of 70% ethanol somehow decreases with exposure (treatment) time where the number of clean and live explants decrease with increasing exposure time. This decreasing effectiveness is believed to be linked to damages of tissues due to the phytotoxicity of the alcohol [32]. But, other researchers reported about the effectiveness of 70% ethanol (15 sec) in combination with HgCl_2_ in sterilizing ginger explants for micropropagation [13, 20]. The use of fungicide and bactericide detergents like RBK in combination with other agents is a common practice for effective preliminary (or surface) sterilization of explants for *in vitro* propagation of many species [23–25]. However, the present study showed that RBK (0.25% w/v) was not effective at 30, 45, or 60 min exposure time. In fact, increasing exposure time caused more contaminated explants. Similar observations were reported with Bavistin and Carbendazim disinfectants by other researchers [32, 34]. Yet, other researchers reported good sterilizing capacity of Bavistin in cleaning ginger explants for tissue culture [35, 36].

### 3.2. The Efficacy of Antibiotics in Controlling Bacterial Contamination

Microbial contamination is another critical problem in plant *in vitro* clonal propagation [37]. It causes big loss to tissue culture and micropropagation companies by affecting the efficiency of propagation and the quality of the outputs [38–40]. This is because customary cultural and management practices are not always sufficient to control microbial contamination of tissue culture facilities and *in vitro* cultures [17]. Thus, the use of antibiotics is highly recommended to alleviate the problem [41]. The sources of contamination of *in vitro* cultures are diverse. Among other things, explants collected from field-grown plants acquire the contaminants from soil [42, 43] or water [44]. It becomes customary to devise procedures to clean the explants off microbial contaminants. The growth media may also be treated with antibiotics to control the growth of bacteria and fungi when the problem of contamination cannot be mitigated by other procedures [45].

The present study tested the effectiveness of four broad-spectrum antibiotics in controlling bacterial contamination of *in vitro* growth media of shoot tip explants of the Deribo cultivar. First, the efficacies of the antibiotics streptomycin, kanamycin, gentamicin, and cefotaxime were tested singly or in combination of two at three different concentrations. The efficacies of each of the antibiotics were tested at the 50, 100, and 200 mg/L concentrations. Streptomycin, kanamycin, and gentamicin were ineffective at all concentrations yielding 0 to 27% clean microshoots. However, cefotaxime was found to be effective at all concentrations yielding significantly higher mean number of clean microshoots (4.33 ± 1.15 to 4.67 ± 0.58 or 87 to 93%) (Table 2; *p* ≤ 0.05). Unfortunately, except at 50 mg/L, the other concentrations caused the death of 40 to 47% of the microshoots.

The use of single antibiotics may not be effective in controlling bacterial contamination of the explants or the media. Thus, the use of a combination of antibiotics is advisable. The present study also examined the efficacies of the possible combinations of two of the four antibiotics in controlling bacterial contamination. Of all the treatments, the combination of cefotaxime and streptomycin at 25 to 100 mg/L yielded higher mean number of clean microshoots (4.33 ± 1.15 to 4.67 ± 0.57 or 87 to 93%) (Table 3; *p* ≤ 0.05). Interestingly, three pairs of antibiotics, namely, streptomycin and kanamycin, kanamycin and cefotaxime, and kanamycin and gentamicin failed to produce any clean microshoots.

Antibiotics are often used in controlling bacterial and fungal contamination of *in vitro* explants and growth media. Unfortunately, the antibiotics could damage essential tissues of explants or kill the explants altogether. Hence, identification of the best antibiotics (or combination of antibiotics), the establishment of the optimum concentration, and the determination of the best exposure time become necessary. Our study showed that only 50 mg/L cefotaxime was effective in producing 87% clean and live explants while higher concentrations were phytotoxic. Likewise, the application of cefotaxime in combination with streptomycin (at 25 mg/L) yielded 87% clean explants. Similar findings were reported with cefotaxime alone and in combination with streptomycin sulphate in controlling bacterial contamination in ginger shoot explant at 50 to 200 mg/L [15]. The application of cefotaxime singly or in combination with other antibiotics was also reported to be effective in controlling bacterial contamination in explants and culture media [41].

Streptomycin plus gentamicin, as well as cefotaxime and gentamicin, yielded some clean explants. It is useful to note that cefotaxime did not produce clean explants when combined with kanamycin. The antibiotic combinations that did not produce clean microshoots caused the death of many microshoots. Streptomycin in combination with cefotaxime at 25 mg/L caused the lowest death of explants (Table 3). Other studies showed best results with gentamicin [46, 47] and streptomycin sulphate [15, 34, 48]. Gentamicin and streptomycin alone or in combination with each other and with the other antibiotics were ineffective. Limited efficacy of streptomycin even at higher dose was also reported elsewhere [41]. Contrarily, Mengs [37] reported 75% clean explants of ginger with 100 mg/L of streptomycin but with 47% survival rate.

### 3.3. Effects of the Antibiotics on Shooting Performance of Ginger Explants

The principal goal of *in vitro* regeneration of plants is to produce mass of healthy and vigorous plantlets quickly. Therefore, any treatment of tissue culture media and/or the plantlets aiming at controlling microbial contaminants should not interfere with the plant growth regulators, the MS media, and the structure and function of the plantlets (i.e., the quality of the plantlets). Chemical agents and antibiotics that adversely affect *in vitro* regeneration are useless. The present study looked into the effects of the four antibiotics (alone and in combination) on the shooting performances of shoot tip explants of the Deribo ginger cultivar.

The study on the effects each antibiotics showed that the highest mean shoot number per explant was observed with 50 mg/L cefotaxime (7.10 ± 0.36; *p* ≤ 0.05). The next highest mean shoot number (2.86 ± 0.59) was observed with the same antibiotics at 100 mg/L (Table 4). All other treatments yielded a mean shoot number of 1.00 (in control) to 2.46 ± 0.16 (with 200 mg/L cefotaxime). Likewise, cefotaxime yielded better mean shoot length ranging from 2.02 ± 0.23 cm (with 200 mg/L) to 4.20 ± 0.26 cm (with 50 mg/L). With the exception of streptomycin yielding a mean shoot length of 2.50 + 0.25 cm, the rest of the treatments produced a mean shoot length of 0.50 to 1.68 cm. However, some of the microshoots produced in the cefotaxime-treated media were getting yellowish. The more the concentration of the antibiotics gets, the more the plantlets become yellowish.

Likewise, the test on the effects of combinations of the antibiotics showed that cefotaxime in combination with streptomycin (at 25 mg/L) produced the highest mean shoot number (7.51 ± 0.27) per explant. The second highest mean shoot numbers were recorded in shooting media treated with cefotaxime plus gentamicin (3.10 ± 0.17) and cefotaxime plus streptomycin (3.01 ± 0.27) (Table 5). By the same token, statistically highest mean shoot length values were recorded in treatments with cefotaxime plus streptomycin (3.56 ± 0.17 cm) and cefotaxime plus gentamicin (2.27 ± 0.25 cm). The cefotaxime plus streptomycin caused the yellowing of 7 to 36% of plantlets. Interestingly, when the plantlets were subcultured into fresh, antibiotics-free media, they grew normal and turned green. Similar observation was reported elsewhere [41].

We observed that 50 mg/L cefotaxime and 25 mg/L cefotaxime plus streptomycin resulted in the best shooting performance as expressed in terms of mean number of shoots per explant and mean shoot length of the explants. Higher concentrations were not only less effective but also more toxic. Similar findings were reported with ginger [15]. The yellowing of microshoots observed in our study was also reported elsewhere [41]. Interestingly, the microshoots turned green and grew normal upon sub-culturing. Some researchers have formulated an effective cocktail of antibiotics comprising of erythromycin, streptomycin, carbenicillin, penicillin, streptomycin, amphotericin B, and NaOCl that was effective in controlling contamination but failed to satisfy the nonphytotoxic criterion [45]. This implies that formulations that can effectively control contaminations are not always suitable in enhancing micropropagation.

## 4. Concluding Remarks

Ginger is one of the most important global commodities that bring millions of dollars in revenue to many countries of the world including Ethiopia. The Ethiopian ginger cultivar Deribo possesses some qualities that made it more preferred in the European market. The variety is cultivated by small-scale farmers in many parts of southern and western Ethiopia with no inputs of advanced technology and scientific agronomic practices. This led to the devastation of the ginger growing areas by the introduction and spread of ginger wilt bacteria since 2014. The adoption of relevant knowledge and technology for mitigating the problem and promoting the sector is long overdue. This paper presented the results of a scientific exercise aiming at developing protocols of sterilizing ginger shoot tip explants and controlling bacterial contamination of *in vitro* ginger micropropagation media. The exercise yielded outstanding results towards developing an optimized and refined *in vitro* micropropagation protocol of the Deribo ginger cultivar. Future studies need to aim at establishing the best procedure of sterilizing ginger shoot tip explants and controlling bacterial contaminants of the explants and *in vitro* micropropagation media with minimum or no phytotoxicity to the explants and plantlets generated by the micropropagation process. Many more antibiotics and combinations have to be tested in search of less or nonphytotoxic formulations.

## Figures and Tables

**Table 1:** Effect of surface disinfectants on contamination and survival of shoot tip explants of ginger (*Z. officinale* Rosc).

Treatment		No. of clean explants		No. of survived explants	
Disinfectant	Time (in min)	Mean (SD)	%	Mean (SD)	%
RBK (0.25% w/v)	30	4.33 (0.58)^c^	43	5.68 (0.58)^b^	57
	45	4.00 (1.00)^c^	40	4.68 (0.58)^bc^	47
	60	3.33 (0.58)^c^	33	5.68 (0.58)^b^	57

NaOCl (0.50% v/v)	10	3.67 (0.58)^c^	37	3.68 (0.58)^c^	37
	20	8.00 (1.73)^a^	80	7.33 (0.58)^a^	73
	30	6.33 (0.58)^b^	63	7.00 (1.00)^a^	70

Ethanol (70% v/v)	5	4.66 (0.58)^c^	47	7.33 (0.58)^a^	73
	10	3.66 (0.58)^c^	37	7.00 (1.00)^a^	70
	15	4.33 (1.15)	43	6.33 (0.58)^ab^	63

Means in the same column with different letters are statistically significantly different at *p* ≤ 0.05; SD: standard deviation.

**Table 2:** The effects of antibiotics on the i*n vitro* bacterial contamination of microshoots of ginger (*Z. officinale* Rosc).

Treatment	mg/L	No. of clean plantlets		No. of killed explants	
		Mean (SD)	%	Mean (SD)	%
Control	0	0.00 (0.00)^c^	0	4.00 (0.00)^a^	80

Streptomycin	50	0.00 (0.00)^c^	0	1.00 (0.00)^c^	20
	100	0.33 (0.57)^bc^	7	2.00 (1.00)^b^	40
	200	1.33 (1.52)^b^	27	2.67 (1.15)^a^	53

Kanamycin	50	0.00 (0.00)^c^	0	0.33 (0.57)^c^	7
	100	0.33 (0.57)^bc^	7	1.00 (0.00)^bc^	20
	200	0.67 (0.57)^bc^	13	3.33 (1.52)^a^	67

Gentamicin	50	0.00 (0.00)^c^	0	1.67 (0.57)^bc^	33
	100	1.00 (0.00)^bc^	13	2.00 (1.00)^b^	40
	200	1.33 (0.57)^b^	27	3.00 (1.00)^a^	60

Cefotaxime	50	4.33 (1.15)^a^	87	0.00 (0.00)^c^	0
	100	4.33 (0.57)^a^	87	2.00 (1.00)^b^	40
	200	4.67 (0.57)^a^	93	2.33 (0.57)^b^	47

Means in the same column with different letters are statistically significantly different at *p* ≤ 0.05; SD: standard deviation.

**Table 3:** Effects of antibiotic combinations on the *in vitro* bacterial contamination of ginger (*Z. officinale* Rosc).

Treatment	mg/L	No. of clean plantlets		No. of killed explants	
		Mean (SD)	%	Mean (SD)	%
Control		0.00 (0.00)^f^	0	4.00 (0.00)^ab^	80

Streptomycin + kanamycin	25	0.00 (0.00)^f^	0	1.33 (1.15)^cd^	27
	50	0.00 (0.00)^f^	0	4.00 (1.00)^ab^	80
	100	0.00 (0.00)^f^	0	3.67 (1.50)^ab^	73

Streptomycin + gentamicin	25	0.67 (0.57)^ef^	13	1.00 (1.00)^cd^	20
	50	1.33 (0.57)^e^	20	1.33 (0.57)^cd^	27
	100	1.67 (1.15)^de^	33	2.67 (1.52)^bc^	53

Streptomycin + cefotaxime	25	4.33 (1.15)^b^	87	0.67 (0.57)^cd^	13
	50	4.33 (0.57)^b^	87	1.33 (1.15)^cd^	27
	100	4.67 (0.57)^a^	93	1.67 (1.15)^cd^	33

Kanamycin + cefotaxime	25	0.00 (0.00)^f^	0	4.67 (0.57)^a^	93
	50	0.00 (0.00)^f^	0	2.67 (1.15)^bc^	53
	100	0.00 (0.00)^f^	0	3.33 (1.52)^ab^	67

Kanamycin + gentamicin	25	0.00 (0.00)^f^	0	3.00 (1.00)^bc^	60
	50	0.00 (0.00)^f^	0	3.33 (0.57)^ab^	67
	100	0.00 (0.00)^f^	0	3.67 (0.57)^ab^	73

Cefotaxime + gentamicin	25	2.00 (1.00)^de^	40	2.00 (1.00)^cd^	40
	50	2.33 (0.57)^cd^	47	1.67 (1.15)^cd^	33
	100	3.00 (1.00)^c^	60	2.67 (0.57)^bc^	53

Means in the same column with different letters are statistically significantly different at *p* ≤ 0.05; SD: standard deviation.

**Table 4:** Effects of single antibiotics on shooting performance of ginger (*Z. officinale* Rosc).

Treatment	mg/L	Mean (SD) shoot number	Mean shoot length (cm)	(%) Color change
Control	0	1.00 (0.00)^e^	0.50 (0.00)^e^	0

Streptomycin	50	2.25 (0.25)^cd^	2.50 (0.25)^b^	0
	100	2.19 (0.17)^cd^	1.68 (0.06)^cd^	0
	200	1.11 (0.19)^e^	0.55 (0.09)^e^	0

Kanamycin	50	1.63 (0.15)^d^	0.92 (0.06)^e^	0
	100	1.50 (0.25)^d^	0.75 (0.13)^e^	0
	200	1.08 (0.14)^e^	0.51 (0.02)^e^	0

Gentamicin	50	1.94 (0.48)^d^	1.40 (0.08)^d^	0
	100	1.89 (0.19)^d^	1.00 (0.33)^de^	0
	200	1.72 (0.25)^d^	1.20 (0.32)^de^	0

Cefotaxime	50	7.10 (0.36)^a^	4.20 (0.26)^a^	13
	100	2.86 (0.59)^b^	2.86 (0.55)^b^	29
	200	2.49 (0.16)^bc^	2.02 (0.23)^c^	38

Means in the same column with different letters are statistically significantly different at *p* ≤ 0.05; SD: standard deviation.

**Table 5:** Effects of antibiotic combinations on the shooting performance of ginger (*Z. officinale* Rosc).

Treatment	mg/L	No. of shoots/explant	Length of shoots (cm)
		Mean (SD)	Mean (SD)
Control		1.00 (0.00)^e^	0.50 (0.00)^e^

Streptomycin + kanamycin	25	1.60 (0.24)^d^	0.69 (0.20)^de^
	50	0.67 (0.57)^ef^	0.40 (0.36)^e^
	100	0.67 (0.57)^ef^	0.46 (0.39)^e^

Streptomycin + gentamicin	25	1.37 (0.32)^de^	0.71 (0.20)^de^
	50	1.75 (0.25)^d^	0.87 (0.12)^d^
	100	1.41 (0.38)^de^	0.91 (0.14)^d^

Streptomycin + cefotaxime	25	7.51 (0.27)^a^	3.56 (0.17)^a^
	50	3.01 (0.27)^b^	1.97 (0.05)^bc^
	100	2.41 (0.14)^c^	1.62 (0.21)^c^

Kanamycin + cefotaxime	25	0.33 (0.57)^f^	0.16 (0.28)^e^
	50	1.56 (0.19)^d^	0.93 (0.20)^d^
	100	1.49 (0.18)^de^	0.82 (0.16)^de^

Kanamycin + gentamicin	25	1.00 (0.00)^e^	0.60 (0.10)^de^
	50	1.17 (0.28)^de^	0.58 (0.14)^de^
	100	1.33 (0.57)^de^	0.63 (0.11)^de^

Cefotaxime + gentamicin	25	3.10 (0.17)^b^	2.27 (0.25)^bc^
	50	1.91 (0.14)^cd^	1.92 (0.14)^bc^
	100	2.50 (0.17)^bc^	1.78 (0.38)^c^

Means in the same column with different letters are statistically significantly different at *p* ≤ 0.05; SD: standard deviation.

## Data Availability

The datasets used and/or analyzed during the current study are available from the first author on reasonable request.
